# Bipolar disorder with binge eating behavior: a genome-wide association study implicates *PRR5-ARHGAP8*

**DOI:** 10.1038/s41398-017-0085-3

**Published:** 2018-02-02

**Authors:** Susan L. McElroy, Stacey J. Winham, Alfredo B. Cuellar-Barboza, Colin L. Colby, Ada Man-Choi Ho, Hugues Sicotte, Beth R. Larrabee, Scott Crow, Mark A. Frye, Joanna M. Biernacka

**Affiliations:** 1 Lindner Center of HOPE, Mason, OH USA; 2 Department of Psychiatry and Behavioral Neuroscience, University of Cincinnati, Cincinnati, OH USA; 3 Department of Health Sciences Research, Mayo Clinic, Rochester, MN USA; 4 Department of Psychiatry, Universidad Autonoma de Nuevo Leon, Monterrey, Mexico; 5 Department of Molecular Pharmacology and Experimental Therapeutics, Mayo Clinic, Rochester, MN USA; 6 University of Minnesota, Minneapolis, MN USA; 7 Department of Psychiatry and Psychology, Mayo Clinic, Rochester, MN USA

## Abstract

Bipolar disorder (BD) is associated with binge eating behavior (BE), and both conditions are heritable. Previously, using data from the Genetic Association Information Network (GAIN) study of BD, we performed genome-wide association (GWA) analyses of BD with BE comorbidity. Here, utilizing data from the Mayo Clinic BD Biobank (969 BD cases, 777 controls), we performed a GWA analysis of a BD subtype defined by BE, and case-only analysis comparing BD subjects with and without BE. We then performed a meta-analysis of the Mayo and GAIN results. The meta-analysis provided genome-wide significant evidence of association between single nucleotide polymorphisms (SNPs) in *PRR5-ARHGAP8* and BE in BD cases (rs726170 OR = 1.91, *P* = 3.05E-08). In the meta-analysis comparing cases with BD with comorbid BE vs. non-BD controls, a genome-wide significant association was observed at SNP rs111940429 in an intergenic region near *PPP1R2P5* (*p* = 1.21E-08). *PRR5-ARHGAP8* is a read-through transcript resulting in a fusion protein of *PRR5* and *ARHGAP8*. *PRR5* encodes a subunit of mTORC2, a serine/threonine kinase that participates in food intake regulation, while *ARHGAP8* encodes a member of the RhoGAP family of proteins that mediate cross-talk between Rho GTPases and other signaling pathways. Without BE information in controls, it is not possible to determine whether the observed association reflects a risk factor for BE in general, risk for BE in individuals with BD, or risk of a subtype of BD with BE. The effect of *PRR5-ARHGAP8* on BE risk thus warrants further investigation.

## Introduction

Bipolar disorder (BD) and binge eating behavior (BE) co-occur more often than expected by chance^1–3^. Estimated to occur in 4.5% of the United States general population, BE has been reported to occur in over 25% of individuals with BD^1,2^. Defined as eating an unusually large amount of food in a discrete time period with a sense of loss of control over the eating, BE is a trans-diagnostic feature of eating disorders: it is a defining symptom of bulimia nervosa and binge eating disorder, and may also occur in anorexia nervosa. BD with comorbid BE is associated with greater psychiatric and general medical burden than BD without BE, and has been proposed to be an important clinical BD subtype^4^. Additionally, each condition is heritable, with heritability estimated at 60–85% for BD and 46–74% for BE^5–9^. Indeed, parental BD is a risk factor for eating disorders, including those with BE, in offspring^10^. Few studies, however, have evaluated the genetic architecture of the co-occurrence of BD and BE. Genetic studies of specific BD sub-phenotypes may help uncover some of the unknown inheritance of BD. In an earlier study using data from the Genetic Association Information Network (GAIN) study of BD, we conducted a genome-wide association (GWA) analysis of BD subtypes defined by the presence or absence of a history of BE and a case-only analysis comparing BD subjects with and without BE history^11^. In that study, no associations were statistically significant at the genome-wide level.

In this study we perform similar analyses in an independent sample of BD patients from the Mayo Clinic Biobank, use the new results to evaluate replication of the prior GAIN results, and conduct a meta-analysis of the two studies, making this the largest GWA study (GWAS) of BE in BD to date. To determine if the GWAS results were enriched for biologically relevant pathways, we then conduct gene-set and network analysis. We also explore specific gene regions previously implicated in BE or related phenotypes.

## Methods

### Study participants from GAIN

We previously performed GWA analyses of BE using data from BD cases and controls collected by the Bipolar Disorder Genome Study Consortium, part of GAIN^11^, accessed using dbGaP^12^. Subjects included 1001 European American bipolar cases and 1034 mentally healthy European American controls. BD cases met DSM-IV criteria for bipolar I disorder or schizoaffective disorder determined by psychiatric interviews based on the Diagnostic Interview for Genetic Studies (DIGS) 2, 3, or 4, which included the question, “has there ever been a time in your life when you went on food binges (i.e., rapid consumption of a large amount of food in a discrete period of time, usually less than two hours)?”. Based on answering this question (yes/no), BD cases were classified as having BE (*N* = 206) or not having BE (*N* = 723). BE data was not available for controls.

### Study participants from Mayo Clinic Biobank

Here, a new sample of BD cases from the Mayo Clinic Bipolar Disorder Biobank^13^, a collaboration between Mayo Clinic and the Lindner Center of HOPE, and controls from the Mayo Clinic Biobank^14^, was used for GWA analyses of BE. Both biobank protocols were approved by an institutional review board, and every participant provided written informed consent to be included in the biobank and future genetic studies. All subjects from the Mayo Bipolar Disorder Biobank had a diagnosis of bipolar I disorder, bipolar II disorder or schizoaffective bipolar type. Entry criteria included age 18 through 80 years and no current suicidal ideation or psychosis. The clinical phenotype of BD subjects was determined with the Structured Clinical Interview for DSM-IV (SCID)^15^ and the Bipolar Biobank Clinical Questionnaire. Current BE was assessed with the Eating Disorder Diagnostic Scale (EDDS)^16–18^. EDDS data was available for 700 of the BD cases passing genotype QC. For the BD plus BE phenotype, subjects had to answer yes on questions 5 and 6 of the EDDS [“During the past 6 months have there been times when you felt you have eaten what other people would regard as an unusually large amount of food (e.g. a quart of ice cream) given the circumstances?” and “During the times when you ate an unusually large amount of food, did you experience a loss of control (feel you couldn’t stop eating or control what or how much you were eating)?”], and thus have any BE within the past six months (*N* = 192 of 700 BD cases with EDDS data).

The control group was selected from the Mayo Clinic Biobank^14^, and excluded potential controls with ICD9 codes for bipolar disorder, schizophrenia, or related diagnoses in their electronic medical record. BE was not assessed among the controls.

### Genotyping, quality control, and imputation

GAIN samples were genotyped on the Affymetrix Genome-Wide Human SNP Array 6.0. Quality control (QC) was performed as described previously^11,19^. After QC, the sample included 1001 European American BD cases and 1034 mentally healthy controls.

Mayo cases and controls (total *N* = 1864) were genotyped using the Illumina HumanOmniExpress platform. We removed SNPs with call rate < 98%, minor allele frequency < 0.01, and those demonstrating departures from Hardy–Weinberg Equilibrium (*P* < 1e-06). We excluded subjects with < 98% call rate, one subject from each related pair (estimated identical-by-descent allele sharing > .2), and subjects of non-European ancestry. We used Structure^20^ to verify European ancestry, and adjusted for remaining population structure using the first four principle components. After QC, the Mayo sample consisted of 969 BD cases and 777 healthy controls without BD.

Genotypes in both the GAIN and Mayo samples were imputed to the 1000 genomes reference panel, as previously described for the GAIN sample^11^. Specifically, SHAPEIT^21^ was used for haplotype phasing, followed by imputation using IMPUTE2.2.2^22^ with the 1000 genome project reference data (phase 1 data, all populations). SNPs with dosage R^2^ < 0.3 or MAF < 0.01 were removed prior to analysis, resulting in more than 8 million SNPs in both datasets.

### Statistical analysis

To identify genetic risk variants of BE in subjects with BD, we previously performed a GWAS of BE in BD GAIN subjects (206 BD subjects with BE vs. 723 BD subjects with no BE)^11^. We now performed a similar GWAS of BE using the Mayo sample (192 BD subjects with BE vs. 508 BD subjects with no BE) and then conducted a meta-analysis of the two studies.

To identify genetic risk variants for a subtype of BD with BE, we also performed a GWAS of BD with comorbid BE, comparing BD cases with BE vs. non-bipolar controls in both the GAIN^11^ (206 BD subjects with BE vs. 1034 non-BD controls) and Mayo (192 BD subjects with BE vs. 777 non-BD controls) samples, and then conducted a meta-analysis of the results.

In each dataset, logistic regression models were fit for each SNP using PLINK version 1.07^19^, adjusted for 4 genomic principle components. SNP values were coded in terms of allele dosage. Fixed-effects meta-analyses were performed using R statistical software version 3.2.3, and *P* < 5E-8 was considered statistically significant.

Finally, we extracted results for several candidate SNPs/genes that were previously implicated in BE or related phenotypes (bulimia nervosa spectrum), including *FTO*, *NT5C1B*, and *HTR2A*^23–25^.

### Follow-up of GWAS results and gene set and network analysis

GWAS results were annotated with NCBI Build 37 to determine location, nearby gene(s), and function. Top results were examined with the UCSC Genome Browser. Potential regulatory function was investigated using ENCODE data via the tools HaploReg (version v4.1)^26^ and RegulomeDB (version 1.1)^27^. Possible expression quantitative trait locus (eQTL) associations between the top SNPs and brain expression were explored with BRAINEAC data (http://www.braineac.org)^28^. For genome-wide significant SNPs, we also explored potential regulatory mechanisms by visualizing 3D compartment organization in the region, based on potential chromatin looping identified with Hi-C analysis, using the bioinformatics tool Juicebox^29,30^. Because large-scale folding organization is thought to be very stable across cell lines^30^, we visualized the 3D structure using the highest resolution dataset (data from the GM12878 lymphoblastoid cell line^30^). Using the GTEX portal V6p^31^, we also examined potential eQTL associations in brain and other tissues.

Case-only and case-control gene-level and gene-set analyses were conducted using MAGMA^32^. Gene-level tests were performed separately by study with multiple regression models using principal components of SNP effects computed from the imputed SNPs with dosage R^2^ > 0.8 and MAF > 0.01. Gene-level meta-analysis was performed for 15,420 genes and utilized in the evaluation of 186 KEGG pathways (http://www.genome.jp/kegg/kegg1.html). Competitive test *p*-values were calculated and corrected for multiple testing via permutation.

Network analysis was performed with Ingenuity Pathway Analysis (IPA; www.ingenuity.com) using SNP *p*-values from the GWAS meta-analyses. SNPs were pre-filtered using LD-clumping with *p* ≤ 0.05, resulting in over 70,000 top SNPs considered for network analysis. SNPs were assigned to the nearest gene using NCBI Build 37, and the top 2000 genes (ranked by minimum SNP *p*-value, all with *p* < 0.0002) were considered ‘significant’ for network analysis. De-novo networks of direct and indirect interactions between significant genes in our data were constructed based on a functional analysis algorithm that utilizes published, peer-reviewed literature within the curated Ingenuity Knowledge Base.

## Results

### GWAS results

**Fig. 1:**
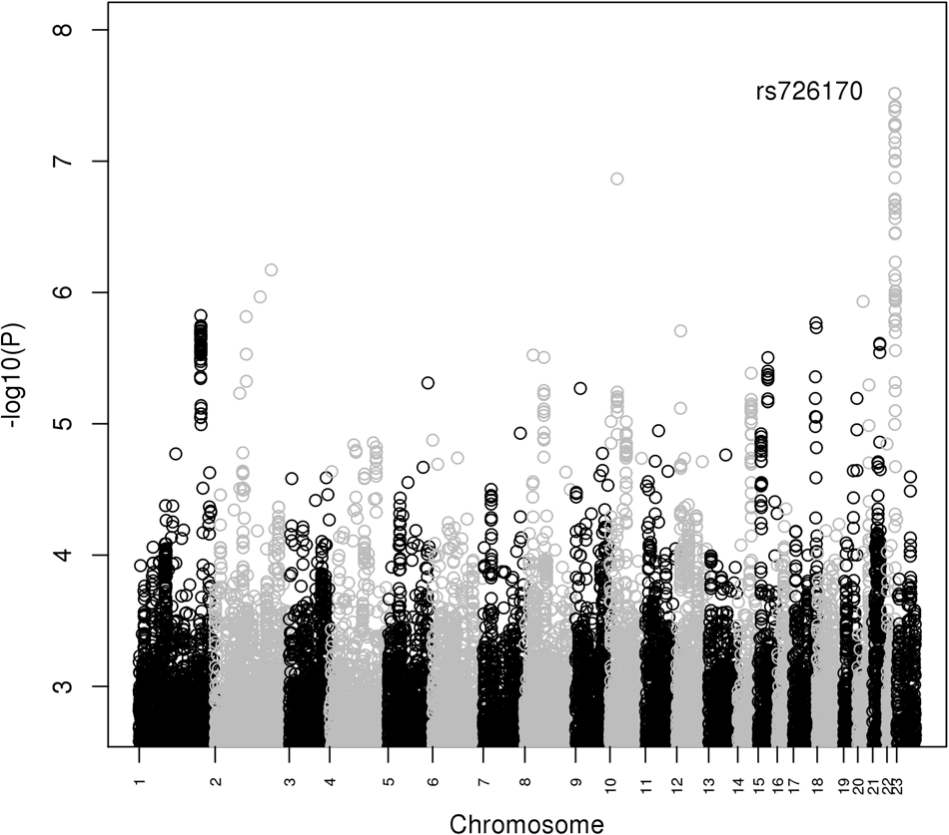
Manhattan plot of GWAS results for the comparison of BD patients with and without the BE behavior. For each SNP, −log10 (*P*-value) is plotted against chromosomal position. The labeled SNP is genome-wide significant at *P* < 5E–8

Supplemental Table 1 shows the sample demographics and supplemental Table 2 and 3 show the top results from the BE case-only GWAS of the GAIN and Mayo data, respectively. The Manhattan plot of the corresponding meta-analysis is shown in Fig. 1, with top results listed in Table 1. The meta-analysis of BE in BD cases provided genome-wide significant evidence for association of BE with a group of SNPs in the *PRR5-ARHGAP8* gene, where the more common allele is associated with reduced risk of BE behavior among BD patients (top SNPs rs726170 and rs8139558, OR = 0.52, *p* = 3.05E-08); the C allele at rs726170 is less common in BD patients with BE (Mayo 0.84, GAIN 0.81) as compared to those without BE (Mayo 0.89, GAIN 0.90). Suggestive evidence of association with rs726170 in *PRR5-ARHGAP8* was observed in our prior analysis of this phenotype in the GAIN data (*p* = 8.0E–08);^11^ the current analysis of the Mayo data replicated this finding (*p* = 0.0046), and the meta-analysis combining the results provided genome-wide significant evidence for association. Additionally, the meta-analysis showed suggestive evidence of association of the G allele at rs7904579 in the *CUBN* gene with BE behavior among BD patients (OR = 1.56, *p* = 1.4E-07), although this association was not genome-wide significant.

**Table 1:** Meta-analysis of BE in BD cases: top results

SNP	Locus	Position	Gene	A1	A2	Mayo A1 freq	GAIN A1 freq	Mayo OR	Mayo P	GAIN OR	GAIN P	Meta-analysis OR	Meta-analysis *p*-value
rs726170	22q13.31	45251811	*PRR5-ARHGAP8*	C	T	0.88	0.88	0.62	0.0046	0.45	8.0E–07	0.52	3.05E–08
rs7904579	10p13	17131753	*CUBN*	G	C	0.35	0.37	1.62	6.9E–05	1.50	4.9E–04	1.56	1.36E–07
rs1950038	2q32.1	184444370	intergenic	T‘	C	0.30	0.30	1.86	2.1E–06	1.36	0.027	1.60	6.73E–07
rs182107583	2q23.3	150531537	LOC101929321 (lncRNA)	A	C	0.96	0.96	0.34	0.0015	0.29	2.0E–04	0.31	1.08E–06
rs76087671	20p11.21	24311177	intergenic	C	T	0.94	0.95	0.61	0.0497	0.32	1.4E–06	0.43	1.17E–06

*Note*: Odds ratio (OR) estimates are presented in terms of A1 vs. A2

**Fig. 2:**
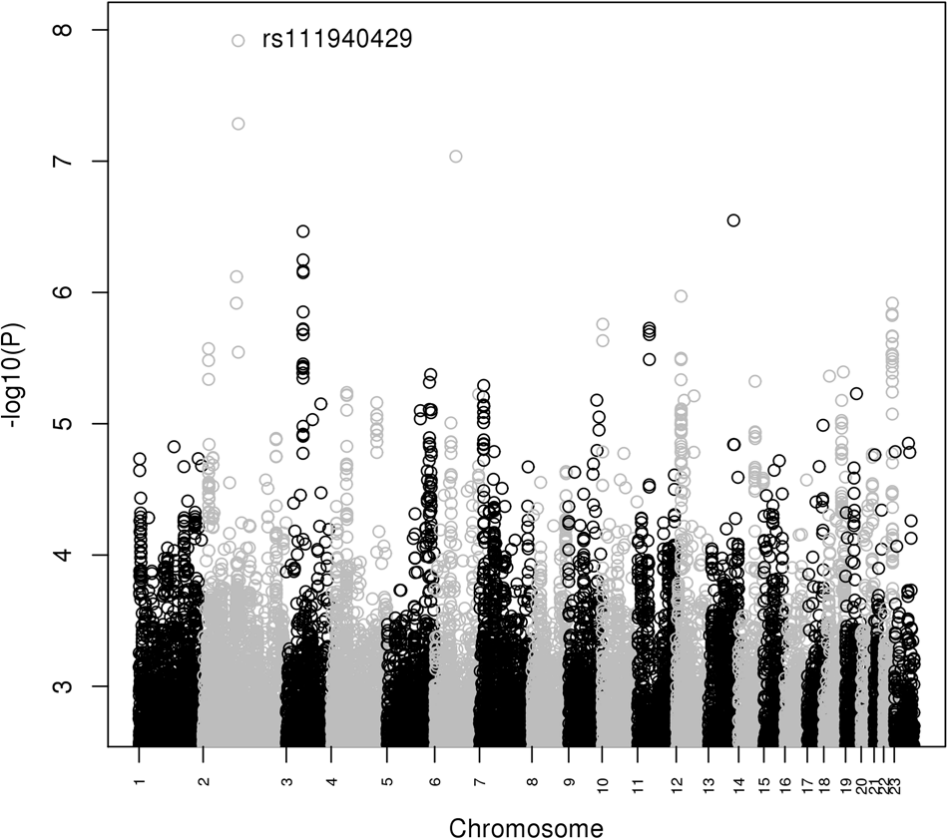
Manhattan plot of GWAS results for the comparison of BD patients with the BE behavior sub-phenotype to controls. For each SNP, −log10 (*P*-value) is plotted against chromosomal position. The labeled SNP is genome-wide significant at *P* < 5E–8

Supplemental Tables 4 and 5 show the top results from GWAS comparing cases with both BD and BE vs. non-BD controls in the GAIN and Mayo data, respectively. The Manhattan plot of the corresponding meta-analysis is shown in Fig. 2, with top results listed in Table 2.The top SNP rs111940429, which is genome wide significant (*P* = 1.2E–08), is in an intergenic region of chromosome 2q12.3, with the closest gene *PPP1R2P5*; the C allele is less common in BD patients with BE (Mayo 0.93, GAIN 0.93) than in controls (Mayo 0.96, GAIN 0.97).

**Table 2:** Meta-analysis of BD with BE vs. controls: top results

SNP	Locus	Position	Gene	A1	A2	Mayo A1 freq	GAIN A1 freq	Mayo OR	Mayo P	GAIN OR	GAIN P	Meta-analysis OR	Meta-analysis *p*-value
rs111940429	2q12.3	107982688	AC096669.1 (lncRNA)	C	T	0.96	0.96	0.34	5.9E–05	0.36	5.2E–05	0.35	1.2E–08
rs17810023	6q14.1	81152773	RP11–250B2.3 (lncRNA)	C	T	0.98	0.98	0.23	0.0011	0.28	2.2E–05	0.26	9.2E–08
rs7337127	13q33.2	105275930	intergenic	C	T	0.87	0.85	0.60	7.2e–04	0.54	1.0E-04	0.57	2.8E–07
rs145763646	3p14.1	66159153	*SLC25A26*	G	A	0.89	0.9	0.55	0.0018	0.40	2.9E-05	0.48	3.4E–07
rs73057489	12p12.3	17523754	intergenic	A	C	0.93	0.93	0.51	0.0011	0.50	2.9E–04	0.50	1.1E–06

*Note*: Odds ratio (OR) estimates are presented in terms of A1 vs. A2.

**Fig. 3:**
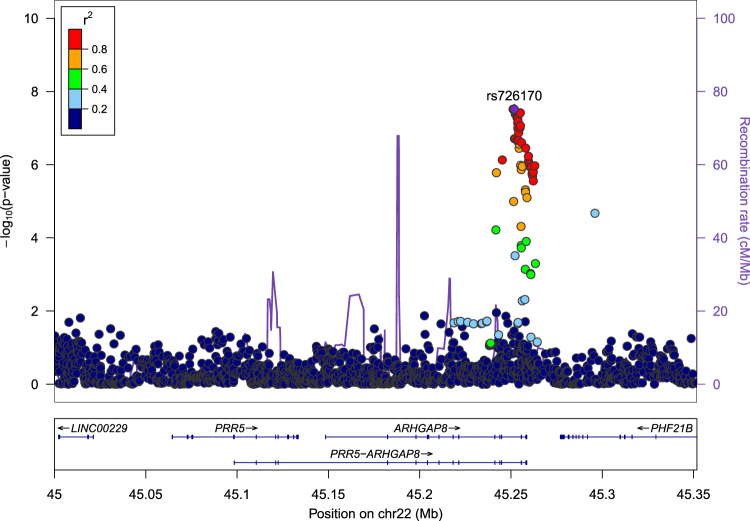
Regional association plot of the *ARHGAP8-PRR5* gene region, for association with BE in cases with BD

### Regulatory analysis results

The genome-wide significant SNP from our case-only meta-analysis, rs726170, is in high linkage disequilibrium (LD) with rs9614952 and rs28439052, which are situated at binding sites for transcription factor *STAT3* (Fig. 3). It is also in complete LD with rs8139558, which is in an enhancer region of *PRR5-ARHGAP8* with H3K4me1 histone mark in adipose cultures^26^. No significant eQTL associations between rs726170 and *PRR5-ARHGAP8* expression were observed in human brain regions studied in BRAINEAC^28^. Using the GTEX portal (version V6p)^31^, rs726170 is a significant eQTL for *ARHGAP8* in tibial nerve (*p* = 3.8e–9), but has no association with expression of the next 5′ gene (*PHF21B*) or the next 3′ gene (*PRR5*).

Supplemental Fig. 1 shows the 3D genomic organization around rs726170. Yellow lines indicate block regions defined by marks of the transcription factor CCCTC-binding factor (CTCF), which delineates heterochromatin from euchromatin. This SNP is in the transition region between two blocks and shows little long range interactions, so it likely affects only the *ARHGAP8* gene or another gene downstream (rather than genes 3′ of *ARHGAP8*), consistent with the GTEX eQTL results. According to ENCODE data, this region is devoid of common histone marks in LCL tissues, with the exception for H3K9me3, normally associated with constitutively repressed genes.

Furthermore, rs111940429 identified in the case-control meta-analysis also shows evidence of regulatory function; specifically, the risk (‘C’) allele in our analysis potentially disrupts the motif for the transcription factor *Fox*^26^.

### Gene-set analysis and network analysis results

MAGMA analyses did not identify any gene sets with statistically significant associations with BE after correction for multiple testing. The top-ranking gene sets in the case-only analysis and in the case-control analysis are shown in Supplemental Table 6 and Supplemental Table 7, respectively.

Constructed IPA networks focus on Neurological Disease, Psychological Disorders, Cellular Development (Supplemental Fig. 2), as well as Hereditary Disorders, Neurological Disease, and Organismal Injury and Abnormalities (Supplemental Fig. 3). Hub molecules with at least 5 connections in Network 1 (Supplemental Fig. 2) include *WWOX*, *CUL3*, *CAND1*, *CDC5L*, and *SMARCA4*. Hub molecules with at least 5 connections in Network 2 (Supplemental Fig. 3) include *TADA2A*, *MED15*, *TCF7L2*, *ATXN1*, and *RBFOX1*.

### BE association with candidate genes

We found no evidence of association of BD with comorbid BE, or BE in BD patients, with a set of SNPs/genes selected based on prior human candidate gene studies (*FTO*, *NT5C1B*, and *HTR2A*). No SNP within these genes was statistically significant after Bonferroni correction for the number of candidate SNPs tested. The top result was rs7330461 in *HTR2A* (uncorrected *p* = 0.003, corrected *p* = 0.90).

## Discussion

In this study, we utilized two BD case-control GWAS datasets to explore genetic risk for the sub-phenotype of BD with comorbid BE. This is the largest GWAS of BE in BD to date, and no GWAS of BE in the general population has yet been published.

We demonstrated that BE among BD patients is associated with variants of the *PRR5-ARHGAP8* gene, a read-through transcript of neighboring genes *PRR5* and *ARHGAP8*. *PRR5* is a circadian clock gene that encodes a subunit of the mammalian target of rapamycin complex 2 (mTORC2) critical for neuronal survival, proliferation, and differentiation^33^, as well as energy balance, obesity, and hyperphagia^34^. *ARHGAP8* encodes a member of the large RhoGAP family of proteins, which are complex tissue-specific molecules regulated by lipid binding, protein–protein interactions, phosphorylation, and other mechanisms^35^. RhoGAPs mediate the cross-talk between Rho GTPases and other signaling pathways, and are fundamental to multiple cellular processes, such as cellular growth, gene transcription, and apoptosis^35^. Read-through transcripts are fusions across adjacent genes resulting in proteins that share sequence identity with each individual gene product^36^. The *PRR5-ARHGAP8* read-through occurs in nature^37^ and is expressed in the brain^31^. Despite the unclear function of the *PRR5-ARHGAP8* fusion protein, *mTORC2* and Rho GTPases interact in different cellular processes, for example in regulating the cytoskeleton^38^, where this fusion protein may play a role.

Further information points to a potential regulatory role of rs726170, although the targets are unclear. This SNP is in high LD with SNPs in binding sites for the transcription factor *STAT3* and an enhancer region in adipose tissue. While we have no evidence of this SNP being an eQTL for either *PRR5* or *ARHGAP8* in brain tissue, the variant is an eQTL for *ARGHAP8* in tibial nerve tissue. Additionally, Hi-C analysis suggested that the 3D chromatin structure of the region prohibits long-range interactions with genes that are upstream of the region, suggesting that the target is either *ARHGAP8* or another gene downstream, rather than *PRR5*. *ARHGAP8* is expressed at low levels in brain and blood, but high levels in gastrointestinal-related tissues. Given that rs726170 was not strongly associated with BD with comorbid BE compared to controls, collectively these results suggest that the identified SNP likely plays a role in BE rather than in BD.

In the meta-analysis of BD cases with BE compared to controls, we identified a genome-wide significant variant in an intergenic region of chromosome 2q12.3, with the nearest gene encoding protein phosphatase 1 regulatory subunit 2 (*PPP1R2*), an inhibitor of the serine/threonine protein phosphatase 1 (PPP1) that binds to the active site of its catalytic subunit. The PPP1C/PPP1R2 complex has been shown to participate in neural functioning (calcium-induced synaptic scaling^39^, memory suppression^40^, and neurodegeneration^41,42^), as well as mitosis and meiosis^43–46^, cell morphology regulation^47^, and cardiac function^48–50^. *PPP1R2* is expressed in GABAergic neurons in the striatum, cortex, and hippocampus^51^.

Our analyses revealed association trends for other genes and pathways that were not significant after multiple testing correction, but may nevertheless reflect important biological mechanisms. For example, the second top association signal in the case-only meta-analysis of BE among BD patients was in the gene encoding cubilin (*CUBN*), a receptor for the intrinsic factor-vitamin B12 complex, which is essential for neurodevelopment; its deficiency has been implicated in various neuropsychiatric diseases such as depression, schizophrenia, dementia, and cognitive impairment^52,53^. In our MAGMA gene-set analysis comparing BD cases with BE to those without BE, the canonical Wnt signaling pathway was a top-ranked pathway; this pathway is critical to neurogenesis, neurodevelopment^54^, mood stabilizer mechanism of action^55^, and energy balance^56^. Application of IPA to our data produced a network that highlighted genes relevant for neurological and psychological disorders, with the effector of the canonical Wnt signaling pathway *TCF7L2*, a gene involved in genetic risk of BD with comorbid obesity^57,58^, as a network hub (Supplemental Fig. 3). Finally, in our gene-set analysis of BD cases with BE compared to controls, the cytokine-cytokine receptor interaction pathway was among the top-ranking pathways; this finding is intriguing considering the role of cytokines in sickness response and the related suppression of appetite and induction of anhedonia. While possibly interesting, these results require further evidence from independent data before their role in binge eating can be established.

Although no prior GWAS of BE have been performed, a GWAS of anorexia nervosa identified a variant in *SOX2-OT*;^59^ another variant in this region, rs4854912, was later confirmed in a GWAS of bipolar patients with either anorexia nervosa or bulimia nervosa compared to healthy controls, in an analysis that included GAIN data^60^. In our Mayo Biobank sample, we observed no evidence of association of this variant with BE among BD cases, nor in the comparison with control subjects (all *p* > 0.50). However, because our phenotypes here focus on BE rather than anorexia nervosa or bulimia nervosa diagnoses, these differences in findings are not surprising. Candidate gene studies have suggested associations between binge eating and *FTO* in adolescents^24^, binge eating and *HTR2A* in young women^23^, and bulimia nervosa spectrum and *NT5C1B* in female twins^25^, but we did not replicate any of these findings.

This study has several limitations. Without BE information in controls, it is not possible to determine whether the observed association with *PRR5-ARHGAP8* reflects risk for BE in general or risk for a subtype of BD with BE. Additionally, BE was defined differently in the two data sets. In GAIN subjects, BE was defined as a lifetime history of food binges, or the rapid consumption of a large amount of food in a discrete period of time, usually less than two hours. In Mayo subjects, BE was defined as eating a large amount of food and having a sense of loss of control in the last six months. Nonetheless, rates of BE behavior were comparable in the two samples. Finally, the sample size was limited, resulting in low power to detect small and even moderate effects. By reducing phenotypic heterogeneity, which tends to result in increased power, we obtained genome-wide significant findings in this relatively small sample. Nevertheless, it is clear that much larger sample sizes are needed to identify further genetic risk factors for BE.

In summary, this study provided genome-wide significant evidence for association of BD with comorbid BE with variants in the *PRR5-ARHGAP8* region, and identified additional regions of interest in the etiology of BD with comorbid BE. To date, BD genetic risk factors have been difficult to replicate^61^, due in part to extensive disease heterogeneity. The present work demonstrates the importance of reducing this heterogeneity by focusing on sub-phenotypes when investigating the genetic risk of BD and its comorbidities. Further population-based studies are needed to untangle whether the observed genetic association is driven by BD, BE, or both, and functional studies will be needed to determine the molecular targets and mechanisms behind the association.

## Data availability

The GAIN dataset (part of Whole Genome Association Study of Bipolar Disorder) used for the analyses described in this manuscript were obtained from the database of Genotypes and Phenotypes (dbGaP) found at http://www.ncbi.nlm.nih.gov/gap through dbGaP accession number phs000017.v3.p1. The GTEx data used for the analyses described in this manuscript were obtained from: http://www.gtexportal.org/home/bubbleHeatmapPage/ARHGAP8 and http://www.gtexportal.org/home/gene/ARHGAP8 on the GTEx Portal accessed on 30 Nov 2016.

## Electronic supplementary material

Supplemental Material
