# Supplementary material for: Bipolar disorder with binge eating behavior: a genome-wide association study implicates PRR5-ARHGAP8
Source: Transl Psychiatry. 2018 Feb 2;8:40. doi: 10.1038/s41398-017-0085-3 (PMC5804024; doi:10.1038/s41398-017-0085-3)
Supplement: Supplementary file 1 — Supplemental Material [file 41398_2017_85_MOESM1_ESM.docx]

**Supplemental Table 1:** Subject demographics

|  | Mayo | | | GAIN | | |
| --- | --- | --- | --- | --- | --- | --- |
|  | BD with BE | BD without BE | Controls | BD with BE | BD without BE | Controls |
| Age, mean(SD) | 40.0 (13.9) | 44.6 (16.1) | 62.2 (15.3) | 38.1 (12.0) | 43.2 (13.0) | 52.1 (19.0)* |
| Sex, Female N (%) | 131 (68.2) | 292 (57.5%) | 390 (50.1) | 135 (65.5) | 337 (46.6%) | 502 (48.5) |
| BP Type, N (%) BP1 or SZA | 117 (60.9) | 343 (67.6%) | NA | 206 (100) | 723 (100%) | NA |

*estimated from categorical data

**Supplemental Table 2:** Results from the BE case-only GWAS of the GAIN data: Top 5 regions after LD clumping with R^2^=0.5

| SNP | Chr | Position | A1 | A2 | A1 freq | OR | P | Nearest gene |
| --- | --- | --- | --- | --- | --- | --- | --- | --- |
| rs8139558 | 22 | 45251169 | C | T | 0.88 | 0.45 | 8.25E-07 | ARHGAP8//PRR5-ARHGAP8 |
| rs140442098 | 1 | 105463337 | T | C | 0.96 | 0.16 | 8.27E-07 | CDK4PS |
| rs76087671 | 20 | 24311177 | C | T | 0.95 | 0.32 | 1.39E-06 | FLJ33581 |
| rs2634806 | 2 | 36245072 | A | C | 0.31 | 1.92 | 1.72E-06 | RPL21P36 |
| rs13037732 | 20 | 43293270 | G | T | 0.92 | 0.41 | 2.23E-06 | LOC79015 |

**Supplemental Table 3:** Results from the BE case-only GWAS of the Mayo data: Top 5 regions after LD clumping with R^2^=0.5

| SNP | Chr | Position | A1 | A2 | A1 freq | OR | P | Nearest gene |
| --- | --- | --- | --- | --- | --- | --- | --- | --- |
| rs9301529 | 13 | 86913029 | C | A | 0.11 | 2.42 | 2.03E-06 | DDX6P2 |
| rs1950038 | 2 | 184444370 | T | C | 0.30 | 1.86 | 2.08E-06 | LOC644877 |
| rs61966535 | 13 | 81023585 | A | G | 0.93 | 0.35 | 2.13E-06 | SPRY2 |
| rs72893792 | 6 | 79943990 | G | A | 0.92 | 0.37 | 2.89E-06 | HMGN3/ LOC100288198 |
| rs2347598 | 5 | 151255149 | T | C | 0.95 | 0.28 | 3.37E-06 | GLRA1/TRNAQ53P |

**Supplemental Table 4:** Results of the GWAS of BD with BE vs. controls in the GAIN data: Top 5 regions after LD clumping with R^2^=0.5

| SNP | Chr | Position | A1 | A2 | A1 freq | OR | P | Nearest gene |
| --- | --- | --- | --- | --- | --- | --- | --- | --- |
| rs10225746 | 7 | 9054565 | G | A | 0.99 | 0.13 | 7.37E-08 | RPL9P19 |
| rs13223076 | 7 | 9709384 | G | A | 0.89 | 0.45 | 1.71E-07 | PER4 |
| rs13233490 | 7 | 9070269 | G | C | 0.99 | 0.16 | 3.88E-07 | RPL9P19 |
| rs4665788 | 2 | 21188488 | T | C | 0.27 | 1.78 | 4.93E-07 | APOB |
| rs35160338 | 7 | 9711911 | A | G | 0.97 | 0.22 | 5.83E-07 | PER4 |

**Supplemental Table 5:** Results of the GWAS of BD with BE vs. controls in the Mayo data: Top 5 regions after LD clumping with R^2^=0.5

| SNP | Chr | Position | A1 | A2 | A1 freq | OR | P | Nearest gene |
| --- | --- | --- | --- | --- | --- | --- | --- | --- |
| rs74468225 | 8 | 24995093 | G | A | 0.96 | 0.24 | 1.51E-07 | LOC100421161 |
| rs2172562 | 15 | 82185894 | T | A | 0.77 | 0.53 | 1.25E-06 | LOC100288241 |
| rs10415562 | 19 | 14910573 | C | T | 0.24 | 0.46 | 1.40E-06 | OR7C1/ LOC100129118 |
| rs73117978 | 20 | 42197510 | G | A | 0.90 | 0.41 | 2.01E-06 | SGK2 |
| rs1664740 | 5 | 3555933 | C | T | 0.69 | 0.57 | 2.55E-06 | LOC285577 |

**Supplemental Table 6**: Gene set analysis results comparing BD cases with BE to those without BE. Results are presented for pathways with nominal p<0.05 ranked by competitive p-value.

| **Gene Set** | **Number of Genes** | **P** | **Corrected P** |
| --- | --- | --- | --- |
| OOCYTE MEIOSIS | 99 | 0.003 | 0.419 |
| WNT SIGNALING PATHWAY | 130 | 0.004 | 0.507 |
| VIBRIO CHOLERAE INFECTION | 47 | 0.005 | 0.528 |
| DILATED CARDIOMYOPATHY | 77 | 0.007 | 0.671 |
| SULFUR METABOLISM | 10 | 0.014 | 0.879 |
| MAPK SIGNALING PATHWAY | 213 | 0.018 | 0.932 |
| ARRHYTHMOGENIC RIGHT VENTRICULAR CARDIOMYOPATHY ARVC | 66 | 0.039 | 0.995 |
| REGULATION OF ACTIN CYTOSKELETON | 178 | 0.042 | 0.996 |

**Supplemental Table 7:** Gene set meta-analysis results comparing BD cases with BE to controls. Results are presented for pathways with nominal p<0.05 ranked by competitive p-value.

| **Gene Set** | **Number of Genes** | **P** | **Corrected P** |
| --- | --- | --- | --- |
| KEGG VIBRIO CHOLERAE INFECTION | 47 | 0.003 | 0.363 |
| KEGG MAPK SIGNALING PATHWAY | 213 | 0.003 | 0.395 |
| KEGG CYTOKINE CYTOKINE RECEPTOR INTERACTION | 215 | 0.005 | 0.541 |
| KEGG SULFUR METABOLISM | 10 | 0.019 | 0.941 |
| KEGG REGULATION OF ACTIN CYTOSKELETON | 178 | 0.027 | 0.978 |
| KEGG AMINO SUGAR AND NUCLEOTIDE SUGAR METABOLISM | 33 | 0.045 | 0.998 |

**Supplemental Figure 1:** 3D chromatin organization around SNP rs726170 at position 45,251,811 in human reference genome GRCh37, provided by Juicebox (based on Rao et al^1^ data on the GM12878 lymphoblastoid cell line). The yellow lines indicate block regions delineated by CTCF marks; rs726170 is in the transition region between two blocks.

**Supplemental Figure 2:** Gene network from broad BE behavior phenotype in the case-only analysis, constructed by IPA.


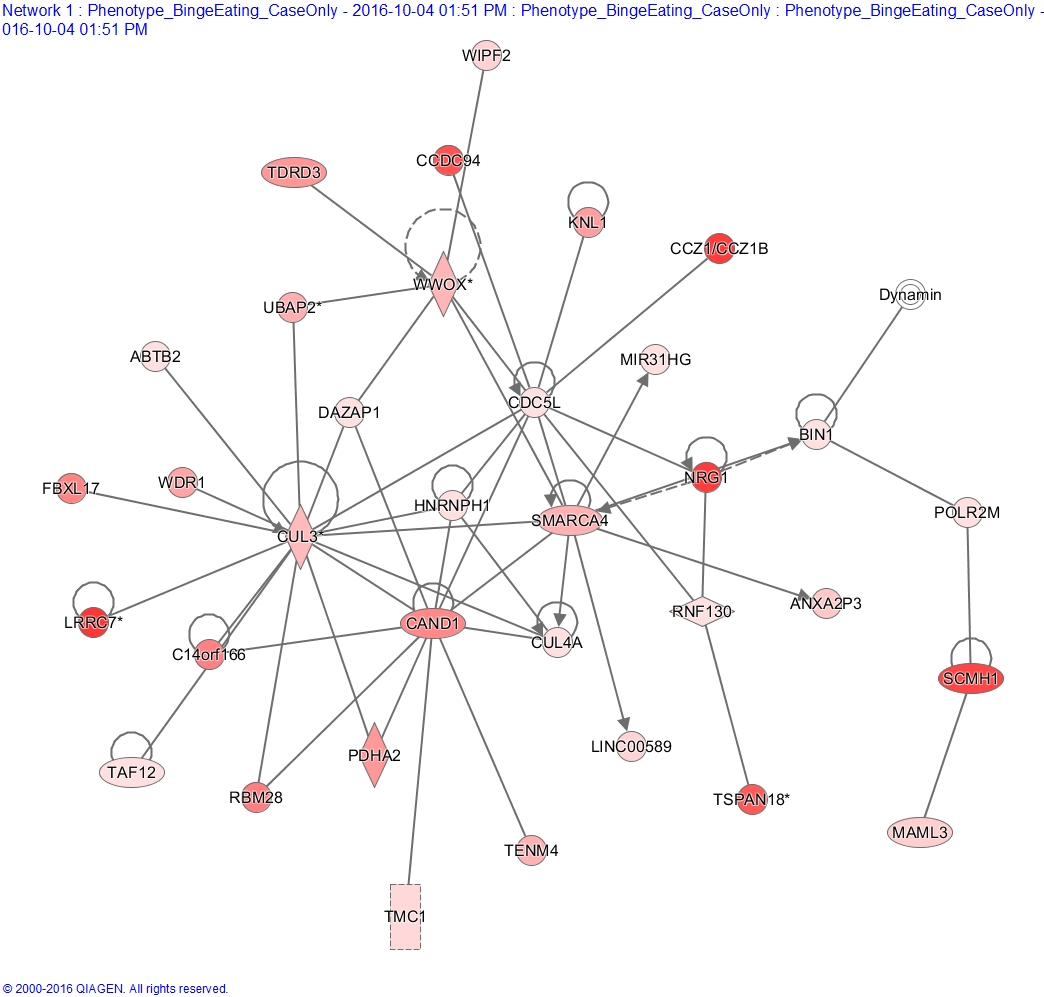


**Supplemental Figure 3:** Gene network from broad BE behavior phenotype in the case-only analysis, constructed by IPA.
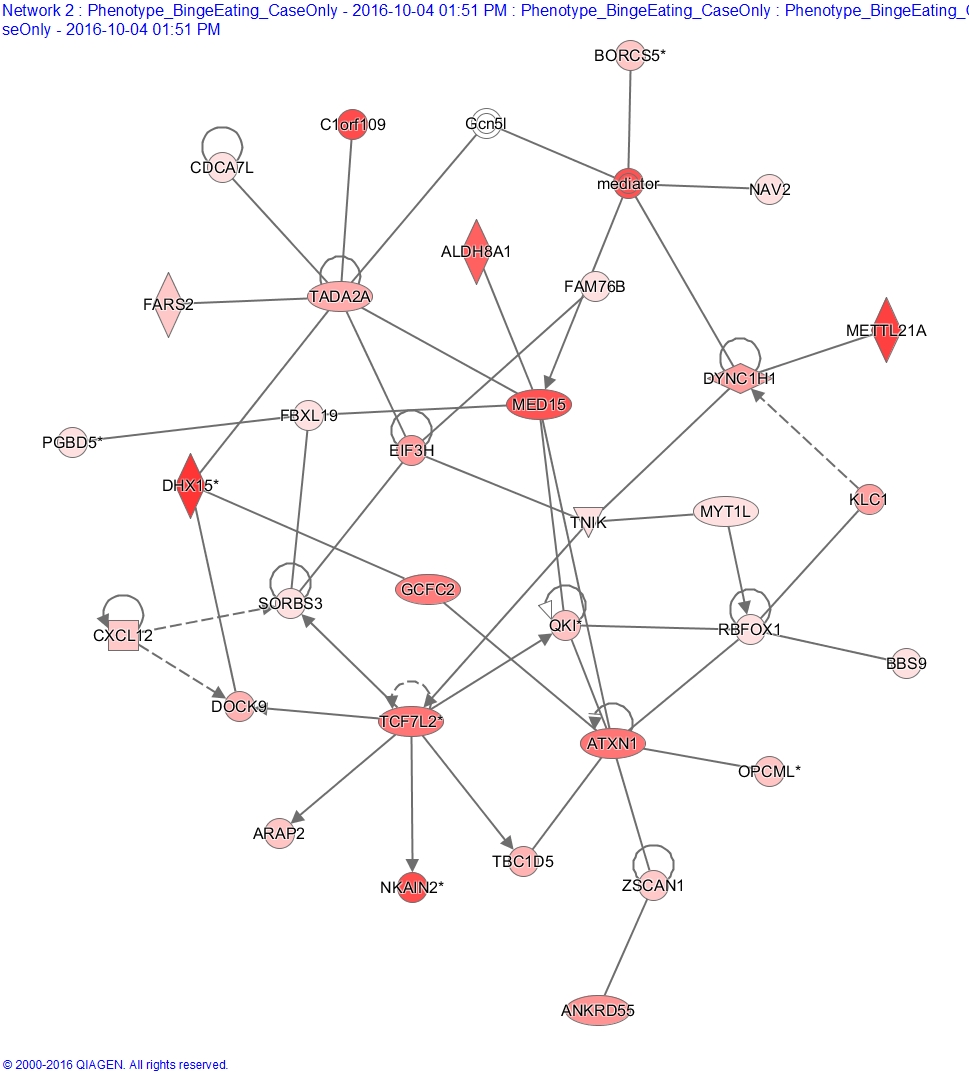


References

1. Rao, S.S., Huntley, M.H., Durand, N.C., Stamenova, E.K., Bochkov, I.D., Robinson, J.T., Sanborn, A.L., Machol, I., Omer, A.D., Lander, E.S., et al. (2014). A 3D map of the human genome at kilobase resolution reveals principles of chromatin looping. Cell 159, 1665-1680.
